# Untangling spider silk evolution with spidroin terminal domains

**DOI:** 10.1186/1471-2148-10-243

**Published:** 2010-08-09

**Authors:** Jessica E Garb, Nadia A Ayoub, Cheryl Y Hayashi

**Affiliations:** 1 Department of Biology, University of California, Riverside, CA 92521, USA; 2 Department of Biological Sciences, University of Massachusetts Lowell, Lowell, MA 01854, USA; 3 Biology Department, Washington & Lee University, Lexington, VA 24450, USA

## Abstract

**Background:**

Spidroins are a unique family of large, structural proteins that make up the bulk of spider silk fibers. Due to the highly variable nature of their repetitive sequences, spidroin evolutionary relationships have principally been determined from their non-repetitive carboxy (C)-terminal domains, though they offer limited character data. The few known spidroin amino (N)-terminal domains have been difficult to obtain, but potentially contain critical phylogenetic information for reconstructing the diversification of spider silks. Here we used silk gland expression data (ESTs) from highly divergent species to evaluate the functional significance and phylogenetic utility of spidroin N-terminal domains.

**Results:**

We report 11 additional spidroin N-termini found by sequencing ~1,900 silk gland cDNAs from nine spider species that shared a common ancestor > 240 million years ago. In contrast to their hyper-variable repetitive regions, spidroin N-terminal domains have retained striking similarities in sequence identity, predicted secondary structure, and hydrophobicity. Through separate and combined phylogenetic analyses of N-terminal domains and their corresponding C-termini, we find that combined analysis produces the most resolved trees and that N-termini contribute more support and less conflict than the C-termini. These analyses show that paralogs largely group by silk gland type, except for the major ampullate spidroins. Moreover, spidroin structural motifs associated with superior tensile strength arose early in the history of this gene family, whereas a motif conferring greater extensibility convergently evolved in two distantly related paralogs.

**Conclusions:**

A non-repetitive N-terminal domain appears to be a universal attribute of spidroin proteins, likely retained from the origin of spider silk production. Since this time, spidroin N-termini have maintained several features, consistent with this domain playing a key role in silk assembly. Phylogenetic analyses of the conserved N- and C-terminal domains illustrate dramatic radiation of the spidroin gene family, involving extensive duplications, shifts in expression patterns and extreme diversification of repetitive structural sequences that endow spider silks with an unparalleled range of mechanical properties.

## Background

There are numerous types of spider silks and each has its own suite of mechanical properties, including exceptional tensile strengths, extensibilities, and toughness [1,2]. This mechanical diversity is associated with the distinct functional demands of the different silk types and largely stems from variation in the molecular composition of the silk proteins [3,4]. An individual spider spins a multitude of silk types, with each type emerging from its own distinctive set of abdominal silk glands. This complex silk machinery enables spiders to utilize task-specific silks (e.g., for web assembly, egg-case construction, prey wrapping, etc.). Every fiber type is composed of one or more spidroin proteins (spidroin = spider fibroin; [5]). Spidroins synthesized by an individual spider are encoded by multiple gene paralogs, the result of gene duplication and divergence events [6-8]. The complement of spidroin paralogs found within a spider genome varies substantially across species from different families [6,7]. Determining the evolutionary relationships of spidroins is therefore an essential step to understanding spider silk diversification.

**Figure 1:**
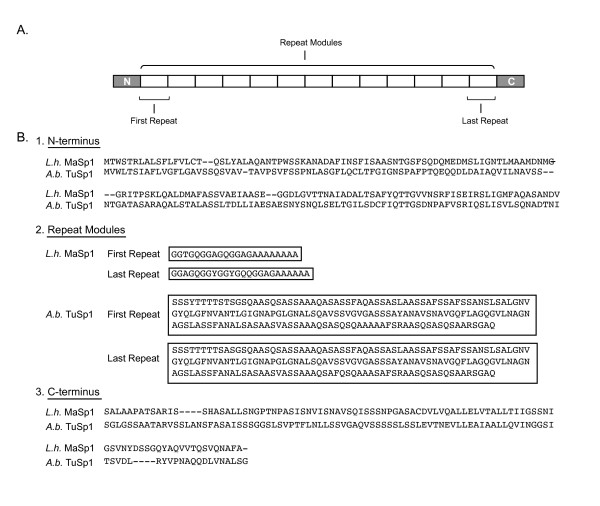
**Spidroin molecular organization and comparison of domain sequences from two paralogs**. A. Schematic of spidroin primary structure showing short, non-repetitive N- and C-terminal domains flanking a long region of tandem sequence repeat modules. B. A comparison of full-length, divergent spidroin paralogs encoding the dragline silk protein MaSp1 from *Latrodectus hesperus *(*L.h*.) [Genbank: EF595246] and the egg-case silk protein TuSp1 from *Argiope bruennichi *(*A.b*.) [Genbank: AB242144], showing their (1) N-terminal domains, (2) the first and last repeat in each sequence and (3) their C-terminal domains. Dashes are alignment gaps. Note the varying repeat sequence length and composition between *L.h*. MaSp1 and *A.b*. TuSp1, in comparison to the high similarity across repeat modules within a spidroin.

Spidroins are typically very large proteins (e.g., > 3000 amino acids, > 200 kDa) and exhibit a polymeric organization, where > 90% of the sequence is composed of highly homogenized tandem repeats [9-12]. Depending on the type of spidroin, these tandem repeats may contain combinations of amino acid sequence motifs that form structural modules such as crystalline beta-sheets, beta turns, or helices, that underlie the mechanical attributes of spider silks [4,13-15]. Flanking a spidroin's long core region of iterated repeats are short, non-repetitive amino (N) and carboxy (C) terminal domains (Figure 1). Sequence conservation of these terminal domains across spidroins, and their presence in silk fibers [16-18], imply they serve some critical role. For example, predicted signal peptides in the N-terminal domain are thought to regulate spidroin secretion from silk gland cells [19-21]; whereas experimental data suggest the N- and C- terminal domains contribute to fiber assembly [22-28].

Reconstructing relationships among spidroins based on their repetitive regions is problematic because their extreme variability in length and sequence identity make them difficult to align [6,7,12]. The high variability observed between spidroin repetitive sequences results from mutations being spread across a gene by concerted evolution involving non-reciprocal recombination among intragenic repeats [10,29]. This scrambling and overwriting of repeated sequences violates assumptions of positional homology implied when they are aligned for phylogenetic construction [30,31]. Thus, despite the repetitive region composing the majority of a spidroin sequence, phylogenetic analyses of the spidroin gene family have relied mostly on the much shorter, more conserved C-terminal domain [6,8,21,32].

Spidroin C-terminal sequences are relatively straightforward to deduce via cDNA cloning and represent the overwhelming majority of existing sequence data available for gene family reconstruction. However, the short C-termini (encoded by ~300 bp) provide limited characters from which to infer evolutionary relationships among paralog lineages that could have arisen 300-400 million years ago [33]. Far fewer spidroin N-terminal sequences are known, due to the difficulties associated with direct N-terminal sequencing of silk proteins and cloning full-length spidroin cDNAs or genomic sequences that can be ~10-15 kb or longer [11,12,20]. The few published N-termini suggest promise as an additional source of phylogenetic characters, because they are approximately 50% longer than C-termini and appear to be more conserved [8,12,20,21,34].

The increasing efficiency of DNA sequencing has enabled us to assemble large-scale collections of expressed sequences (ESTs: Expressed Sequence Tags) from spider silk gland cDNA libraries. Through bioinformatic surveys of these data, we identified 11 more N-terminal spidroin sequences from nine species, nearly doubling the set available for phylogenetic inference. Notably, we report N-terminal spidroin sequences from a broad sampling of spider lineages, spanning > 240 million years of divergence, as well as from a greater diversity of functionally distinct silk proteins. We investigate the utility of these expanded N-terminal data, both separately and in combination with corresponding C-termini for resolving spidroin phylogeny and to trace the evolution of structural motifs that contribute to the extraordinary mechanical performance of spider silks. We also identify conserved sequence features of spidroin N-termini that are likely to be important for the production of native spider silk and the assembly of recombinant silk *in vitro *for biomimetic applications.

## Methods

### cDNA library construction and screening

Silk gland cDNA libraries were constructed from nine spider species representing eight families. Live spiders were anesthetized with CO_2 _and the following silk glands were dissected and then flash frozen in liquid nitrogen: (1) tubuliform glands from *Argiope argentata *(Araneidae); (2) minor ampullate glands from *Latrodectus hesperus *(Theridiidae); (3) flagelliform glands from *Metepeira grandiosa *(Araneidae); (4) large, ampullate shaped glands from *Diguetia canities *(Diguetidae); and combined silk gland tissue from (5) *Agelenopsis aperta *(Agelenidae), (6) *Deinopis spinosa *(Deinopidae), (7) *Uloborus diversus *(Uloboridae), (8) *Kukulkania hibernalis *(Filistatidae), and (9) *Bothriocyrtum californicum *(Ctenizidae). Total RNA was extracted from each tissue type by homogenization in TRIzol (Invitrogen, Carlsbad, CA) and further purified using the RNeasy Mini Kit (Qiagen, Valencia, CA). mRNA was isolated with oligo-(dT)-tagged magnetic beads (Invitrogen). cDNA was synthesized using Invitrogen's SuperScript Choice protocol, starting with the anchored oligonucleotide (dT)_18_V. cDNAs were fractionated by size using ChromaSpin 1000 columns (Clontech, Mountain View, CA), blunt-end ligated into pZErO-2 vector (Invitrogen), and electroporated into TOP10 *Escherichia coli *(Invitrogen). For each species, ~1800 colonies were arrayed and replicated onto nylon filters. Between 400-600 recombinant clones from each library were screened for size by visualization of plasmid DNA with gel electrophoresis using the method of Beuken et al. [35] and inserts ≥ 500 bp were sequenced using T7 or Sp6 primers.

The nylon filters of every cDNA library were screened with γ^32^P labeled oligonucleotide probes encoding poly-alanine (GCDGCDGCDGCDGCDGC) and alternating alanine and glycine (CCWGCWCCWGCWCCWGCWCC), motifs common to many spider silks [6]. We also used the following probes to screen specific libraries: (1) GMWGAWGCRAAWGCCATRTT, (2) CRAYMGMAGATGCRAATGCCAT (1-2 for *Kukulkania hibernalis *and *Diguetia canities*); (3) CGATGCGGCTGCTGCAGA, (4) GCCACGACCGAAGTCTCC, (5) CTGATGGGGTTGCTGTCC, (6) GCCTGGTGCTCTCGCCGT, (7) GCTATTTAGAGAGGGGTTGG, (8) CTGATTGCTGGTTTTGCC, (9) AACCGTTTGGAAATTTTG (3-9 for *Diguetia canities*); (10) CCWCCWGGWCCNNNWCCWCCWGGWCC, (11) CCWGGWCCTTGTTGWCCWGGWCC (10-11 for *Metepeira grandiosa*)*; *and (12) CGATGTGGTGGTAGTTCT, (13) AGCGGATGAGAAGGCACT, (14) GGCACTGGAGAAAGCGCT, (15) ACTDGCTCCBACRCCRAC, (16) GAYTGGCTTGCGGCTTGRCT (12-16 for *Agelenopsis aperta*). Additional probes used in screening libraries from *Argiope argentata *were reported in Garb and Hayashi [29], from *Uloborus diversus *and *Deinopis spinosa *in Garb et al. [32], and *Bothriocyrtum californicum *in Garb et al. [7]. Probe-positive recombinant clones were sequenced with T7 and/or Sp6.

### Characterization of spidroin N- and C-terminal domains

To identify putative spidroin N-terminal domain sequences, silk gland EST sequences were subjected to translated-Blast queries (BlastX; [36]) against the NCBI nr protein database. Collected sequences were also compiled in a private database against which we blasted published spidroin N-terminal sequences. Plasmids containing N-terminal coding regions were digested with restriction enzymes to estimate insert size. The longest clone of each N-terminal type, containing the maximal amount of upstream sequence, was selected for further characterization. These cDNAs were independently sequenced two times in their forward and reverse directions using T7 and Sp6. We also surveyed the literature and searched GenBank databases for published N-terminal sequences.

N-terminal sequences were identified as belonging to established spidroin classes by: 1) the presence of recurring amino acid motifs in adjacent repetitive sequence, diagnostic for particular spidroins (see Gatesy et al. [6]); 2) the silk gland from which they were isolated; and 3) their relationship to published N-termini based on preliminary phylogenetic analyses. Spidroin sequence nomenclature indicates the silk gland type from which it was initially isolated (Ma = major ampullate, Mi = minor ampullate, Tu = tubuliform, Ac = aciniform, or Flag = flagelliform), usually followed by "Sp" for spidroin, and often a number for distinct paralogs (e.g., MaSp1 and MaSp2 were the first two spidroins characterized from major ampullate silk glands). Sequences not readily assigned to these groups were designated by species name followed by "fibroin x", where x is a number identifying a distinct paralog (e.g., *Bothriocyrtum californicum *fibroin 1). However, it should be noted that various authors have occasionally assigned the same protein name to paralogous spidroins (e.g., paralogs from different species have been named "MaSp1").

For new and published spidroin N-terminal sequences, we associated each with its corresponding downstream C-terminal sequence. This is trivial in the four cases where full-length cDNA or genomic sequences have been reported [11,12]. However, the great majority of spidroin sequences represent partial transcripts that span either the N-terminal or C-terminal coding region adjacent to downstream or upstream repetitive sequence, respectively. Because spidroin repetitive sequence is extremely similar across its entire length (e.g., see Figure 1), previous work reporting N-termini have identified their probable downstream C-terminal sequence based on near identity of the adjacent repetitive regions in each [20,21,34]. In this paper, we similarly assigned a corresponding C-terminal sequence to an N-terminal sequence (from the same species) if their repetitive regions were nearly identical. GenBank accession numbers for newly reported and published sequences examined in this study are listed in Table 1.

**Table 1:** Spider fibroin (spidroin) sequences analyzed in this study.

N-terminus	Species	GenBank Accession	Reference	C-terminus Accession	Reference
*B.c*. fibroin1	*Bothriocyrtum californicum *	HM752562	This study	EU117162	[7]
*K.h*. MaSp1	*Kukulkania hibernalis *	HM752563	This study	--	--
*D.c*. MaSp	*Diguetia canities *	HM752564	This study	HM752565	This study
*D.c*. MaSp-like	*Diguetia canities*	HM752566	This study	HM752567	This study
*D.s*. MaSp2	*Deinopis spinosa *	HM752568	This study	DQ399328, DQ399329^a^	[32]
*A.ap*. MaSp	*Agelenopsis aperta *	HM752573	This study	AAT08436	[55]
*U.d*. MiSp	*Uloborus diversus *	HM752574	This study	ABD61597	[32]
*M.g*. MiSp	*Metepeira grandiosa *	HM752575	This study	HM752569	This study
*L.h*. MiSp	*Latrodectus hesperus *	HM752570	This study	HM752571	This study
*A.ap*. TuSp1	*Agelenopsis aperta*	HM752576	This study	HM752572	This study
*A.a*. TuSp1	*Argiope argentata *	HM752577	This study	AY953071	[29]
*A.b*. TuSp1	*Argiope bruennichi *	AB242144	[11]	AB242144	[11]
*N.ct*. TuSp1	*Nephila clavata *	AB218974	[64]	AB218973	[64]
*L.h*. TuSp1	*Latrodectus hesperus *	DQ379383	[18]	AY953070	[29]
*A.t*. MaSp2	*Argiope trifasciata *	DQ059136S1	[20]	DQ059136S2	[20]
*N.c*. MaSp2	*Nephila clavipes *	EU599240	[34]	AY654297	[17]
*N.i*. MaSp2	*Nephila inaurata madagascariensis *	DQ059135	[20]	AF350278	[6]
*N.c*. MaSp1a	*Nephila clavipes *	EU599238	[34]	AY654292	[17]
*N.c*. MaSp1b	*Nephila clavipes*	EU599239	[34]	AY654291	[17]
*L.h*. MaSp1	*Latrodectus hesperus *	EF595246	[12]	EF595246	[12]
*L.h*. MaSp2	*Latrodectus hesperus *	EF595245	[12]	EF595245	[12]
*L.g*. MaSp1	*Latrodectus geometricus *	DQ059133S1b	[20]	DQ059133S2	[20]
*E.a*. MaSp	*Euprosthenops australis *	AM259067	[21]	AJ973155	[65]
*N.c*. Flag	*Nephila clavipes *	AF027972^b^	[19]	AF027973	[19]
*N.i*. Flag	*Nephila inaurata madagascariensis *	AF218623S1	[10]	AF218623S2	[10]
*A.v*. Flag	*Araneus ventricosus *	AY945306	--	AY587193	-

^a^DQ399329 contains the *D.s*. MaSp2a C-terminal sequence and DQ399328 the *D.s*. MaSp2b C-terminal sequence. ^b^DQ059133S1 and AF027972 were edited to include corrections outlined by Rising et al. [21].

N-terminal spidroin sequences were determined by conceptual translation using coding frames determined by BlastX searches. Rising et al. [21] identified the presence of a conserved translation initiation site (Met residue) in N-terminal sequences. Following this finding, we presumed that N-terminal transcripts lacked complete upstream coding information if their sequence did not overlap this region. We subjected the N-termini to SignalP http://www.cbs.dtu.dk/services/SignalP/ analyses, which predict the location of signal peptide cleavage sites. Superimposed Kyte-Doolittle [37] hydropathy plots of the N-termini were made with pepwindowall http://emboss.sourceforge.net/apps/cvs/emboss/apps/pepwindowall.html. N-terminal secondary structures were predicted using the Garnier et al. [38] method implemented in GOR IV http://npsa-pbil.ibcp.fr/cgi-bin/npsa_automat.pl?page=npsa_gor4.html.

### Phylogenetic tree construction

N-terminal and C-terminal amino acid sequences were separately aligned with ClustalW, using default parameters as implemented in MacVector 7.0 (Oxford Molecular Group, Oxford, UK), then the output was refined manually. N-terminal sequences of *Latrodectus geometricus *MaSp1 and *Nephila clavipes *Flag were edited according to Rising et al. [21]. Protein sequence alignments were used to guide an alignment of encoding nucleotides using the program tranalign http://bioweb2.pasteur.fr/docs/EMBOSS/tranalign.html. Phylogenetic analyses were performed with N- and C-terminal alignments for protein and nucleotide sequences separately, and also in a combined analysis, concatenating the N- and C-terminal character matrices. Heuristic parsimony tree searches were conducted with PAUP* 4.0b [39], including 10,000 random taxon addition replicates and tree-bisection-reconnection branch swapping, treating gaps as missing data. Branch support was computed from 1000 bootstrap (BT) replicates, with three random taxon addition replicates per bootstrap replicate. Decay indices of tree nodes were determined using the program TreeRot v3 [40].

Bayesian phylogenetic analyses were also conducted separately for N-termini, C-termini, and the two in combination. Analyses were performed with MrBayes 3.1.2 [41], using the model recommended by ProtTest [42] for separate analyses of protein sequences, or by ModelTest [43] for separate and combined analyses of nucleotides. Combined nucleotide analyses were partitioned by N- and C-terminal domains, using the recommended model for each partition. Combined amino acid N- and C-terminal analyses implemented a "mixed" model, allowing switching between models plus a gamma distribution. Bayesian runs were executed for 5 × 10^6 ^generations, sampling trees every 1000 generations, and continued until split frequencies were below 0.01. Clade posterior probabilities (PP) were computed from a 50% majority-rule consensus of post burn-in trees (25% of each run, totaling 125,000 trees).

**Figure 2:**
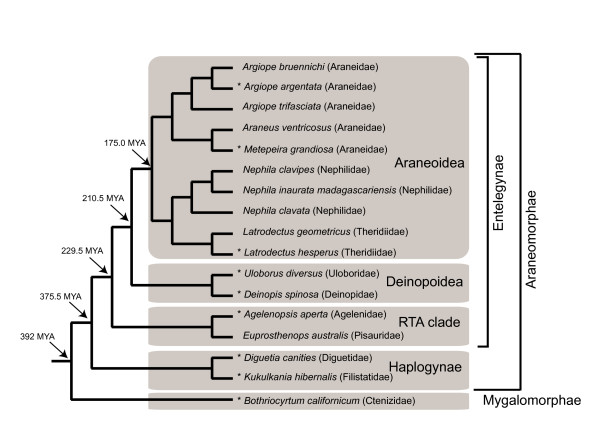
**Phylogeny of species examined in this study**. Tree is a composite of published phylogenies [44-47]. Major lineages and approximate divergence dates estimated by Ayoub and Hayashi [33] are indicated. Asterisks mark species from which we report new N-terminal sequences. Branch lengths are not proportional to time.

Root placement in spidroin gene family trees was estimated with reference to a phylogenetic hypothesis for spider species. This "species tree" included all species from which the analyzed spidroin genes were sampled and is a composite tree, summarized from published phylogenies (Figure 2). Relationships at the family level and above were determined from Coddington et al. [44], relationships among *Nephila *species were from Kuntner et al. [45], relationships within Araneidae are based on Scharff and Coddington [46] and relationships among *Argiope *species were from Elices et al. [47]. Each gene tree was reconciled with the fixed species tree in Notung 2.6 [48], to identify the rooted topology that minimized inferred gene duplications and losses (D/L). Default cost parameters in Notung (duplication = 1.5, loss = 1.0) were used to compute minimal D/L scores and re-root trees.

### Spidroin tree comparisons and character mapping

The resulting N- and C-terminal trees were visually compared to identify any well-supported (BT ≥ 70% or PP ≥ 0.95) but conflicting nodes [49]. Potential conflict between N- and C-termini was also evaluated with the partition homogeneity test (PHT), implemented in PAUP and excluding invariant characters. Null distributions were constructed from 1000 replicate character permutations, with most parsimonious trees for each replicate estimated from 10 random taxon addition replicates. Likelihood tree scores for different N- or C-terminal (and combined) phylogenetic hypotheses were evaluated relative to each dataset. Likelihood values for nucleotide trees were determined in PAUP*, using the recommended substitution model for the given dataset and allowing branch lengths to vary. Significant differences between best and alternative hypotheses were compared using the Shimodaira-Hasegawa (SH) test [50], with full optimization and 1000 bootstrap replicates. Likelihood values of trees derived from amino acid data were determined and compared to alternative trees with the SH test in TreePuzzle 5.2 [51]. For combined parsimony analyses, conflict and congruence for different nodes was also evaluated with Partitioned Branch support [52], computed with TreeRot v3, Hidden Branch Support and Partitioned Hidden Branch Support [53].

The combined amino acid tree was used to map N- and C-terminal domain synapomorphies. Unambiguously optimized apomorphic changes were determined using the "apolist" option in PAUP*, for both ACCTRAN and DELTRAN optimizations, and tracing each onto the combined tree to identify shared derived residues at each tree node. We scored exemplar repeat sequences from each sampled spidroin for the presence of amino acid motifs hypothesized to form specific secondary and tertiary structures [4]. These included poly-alanine, A_n _(four or more contiguous alanines); two or more consecutive glycine-alanine couplets, (GA)_n_; two or more consecutive glycine-serine couplets, (GS)_n_; two or more GPGX_n _repeats, where P = proline and X = an amino acid from a small subset; and two or more tandem arrayed GGX. A_n_, (GA)_n_, and (GS)_n _conform to beta-sheet structures that impart tensile strength, whereas repeating GPGX_n _motifs form beta-turns that confer extensibility, and the (GGX)_n _motif forms a 3_10 _helix [4]. Gain or loss of these motifs at tree nodes was inferred by parsimony ancestral reconstruction using the combined N- and C-terminal domain trees in MacClade 4.0 [54].

## Results

### N-terminal sequence discovery

In total, we sequenced 1,921 silk gland cDNAs from nine spider species. BlastX searches of the resulting EST data identified 30 cDNA sequences containing putative spidroin N-termini. Blastclust analyses http://toolkit.tuebingen.mpg.de/blastclust#, which cluster highly similar sequences, grouped the 30 N-termini into 11 distinct sequence types. Each N-terminal cDNA represented a partial transcript that included some adjacent repetitive sequence. Except for the MaSp1 N-terminal sequence from the nursery web spider, *Euprosthenops australis *[21], previous reports of N-terminal spidroins were from eight species of the spider clade Araneoidea (ecribellate orbweavers; Figure 2). Our new sequences indicate the presence of a similar non-repetitive N-terminal domain in spidroins synthesized by eight additional species, six of which were non-araneoids from the lineages Deinopoidea (*Uloborus *and *Deinopis*), the RTA clade (*Agelenopsis*), Haplogynae (*Kukulkania *and *Diguetia*), and the suborder Mygalomorphae (*Bothriocyrtum*). These included sequences we hypothesize to be upstream of *Agelenopsis aperta *MaSp1 (GenBank accession AAT08436) and *Kukulkania hibernalis *MaSp1 (AAT08433) reported by Tian et al. [55]. In addition, we discovered N-terminal sequences from *Latrodectus*, *Metepeira*, and *Uloborus *for the minor ampullate spidroin MiSp, which constitutes temporary scaffold silk. Fourteen additional published N-terminal spidroin sequences, and one unpublished N-terminal sequence reported in GenBank as a "major ampullate dragline silk protein" (AY945306) but which we attribute to Flag because it flanks repetitive sequence characteristic of Flag spidroins, were included in subsequent analyses (Table 1). We were able to associate N-termini with putative downstream C-termini for all new and published sequences except for *Kukulkania hibernalis *MaSp1, the C-terminus of which is unknown. It was also not possible to determine whether the N-terminal sequence of MaSp2 from *Deinopis spinosa *was upstream of MaSp2a (DQ399329) or MaSp2b (DQ399328) C-terminal sequences, which are very similar to each other and possibly represent allelic variants [32]. For this reason, both MaSp2a and MaSp2b from *D. spinosa *were included in C-terminal analyses.

### N-terminal sequence features

Alignment of the translated sequences showed that five of the 11 newly reported N-termini include the conserved methionine residue identified by Rising et al. [21] as the spidroin translation initiation site (Additional file 1). SignalP 3.0 predicted the presence and location of a signal peptide in nearly all N-terminal sequences, consistent with the targeting of these proteins for entry into the secretory pathway. Three sequences (*A.a*. TuSp1, *A.v*. Flag, and *U.d*. MiSp) were predicted to be non-secretory proteins, a possible artifact of their lack of some upstream sequence. The amino acid sequence motif "TTGXXN" identified by Rising et al. [21] as conserved across spidroin N-termini, does not appear in all of the new sequences we report here (Additional file 1). Of the 168 aligned residues in the N-terminal alignment, only three are present universally in all sequences (Additional file 1). These three residues are the start codon, an aspartic acid (position 70) and a glycine (position 140). However, 39% of all sites contained the same residue in at least half of the sequences. Average pairwise identity across N-terminal amino acid sequences was 37%, and corresponding C-termini shared an average of 35% identity (median pairwise identity for N-termini = 33%, C-termini = 30%). N-terminal regions had proportionately less length variation than C-terminal regions (complete N-terminal sequences ranged from 151-162 amino acids vs. 87-107 amino acids for C-terminal sequences). Superimposed Kyte-Doolittle plots indicated relative similarity in hydropathy profiles across N-termini (Additional file 2), with greatest hydrophobicity occurring in the first 10-20 residues, consistent with predictions that they include signal peptides, followed by alternating hydrophilic and hydrophobic regions. Secondary structural predictions for exemplars of the different N-termini consisted mostly of 4-6 alpha-helices (41-70%) that are connected by short intervening random coils and some extended strands (Additional file 3).

### Phylogenetic analyses

**Figure 3:**
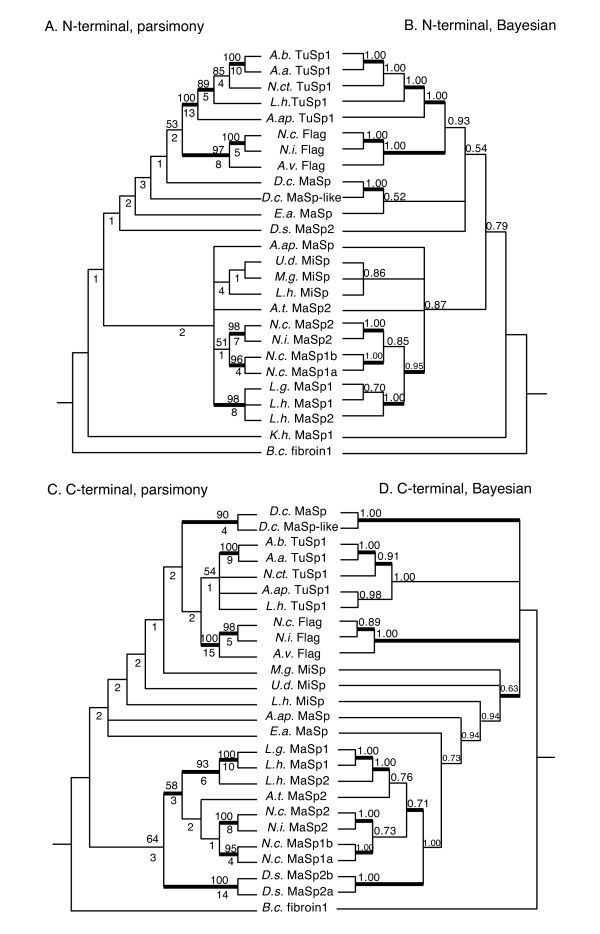
**Phylogenetic trees from separate analyses of spidroin N- and C-terminal domain sequences**. A. N-terminal domain, consensus of 4 most parsimonious trees (MPTs), B. Bayesian consensus tree for N-terminus, C. C-terminal domain, consensus of 8 MPTs, D. Bayesian consensus for C-terminus; A, C: numbers above nodes are bootstrap (BT) values, below nodes decay index, thickened branch supported by ≥ 70 BT in parsimony nucleotide analyses (Additional file 4a, 4c); B, D: numbers above nodes are posterior probability (PP) values, thickened branch supported by ≥ 0.95 PP in Bayesian nucleotide analyses (Additional file 4b, 4d).

At both the nucleotide (nu) and amino acid (aa) level, N-terminal sequences provided more variable (nu = 455, aa = 160) and parsimony informative (PI: nu = 415, aa = 144) characters than C-termini (variable: nu = 300, aa = 102; PI: nu = 283, aa = 96; Table 2). The consistency indices (CI) of most parsimonious trees, were similar for both N-termini and C-termini (e.g., Nterm aa CI = 0.634 vs. Cterm aa CI = 0.643; Table 2). Parsimony analyses of nucleotide data resulted in a single N-terminal tree and two C-terminal trees, whereas Bayesian consensus trees were less resolved (Additional file 4). Nearly all statistically supported nodes in the separate nucleotide trees were supported in the amino acid trees (Figure 3, Additional file 4), and the number of supported nodes did not markedly differ between N- and C-terminal trees (Table 2). Separate N- and C-terminal amino acid trees supported a TuSp1 clade (N-terminal: BT = 100, PP = 1.00; C-terminal: BT = 54, PP = 1.00) and Flag clade (N-terminal: BT = 97, PP = 1.00; C-terminal: BT = 100, PP = 1.00), but relationships among MaSp and MiSp sequences varied. Nevertheless, supported groups of more recently diverged sequences were mirrored in both N- and Cterminal trees (e.g., *D.c*. MaSp + *D.c*. MaSp-like; all *Latrodectus *MaSp sequences).

**Table 2:** Summary statistics for spidroin N- and C-terminal domain character sets.

Data	Chr.	Var.	PI	Len.	CI	RI	MPT	% distance ave (min-max)	branches BT ≥ 70%	branches PP ≥ 0.95	D/L score Parsimony	D/L score Bayesian
N nu	504	455	415	2989	0.367	0.452	1	0.52(0.03-0.66)	10	11	55.5(11/39)	44.5(9/31)
C nu	327	300	283	1877	0.392	0.497	2	0.55(0.01-0.72)	11	13	43(10/28)	50(10/35)
N aa	168	160	144	1223	0.634	0.587	4	0.63(0.01-0.88)	9	11	60(12/42)	53.5(11/37)
C aa	109	102	96	720	0.643	0.635	8	0.65(0-0.90)	9	11	59(12/41)	57(12/39)
N+C nu	831	755	697	4877	0.376	0.459	1	0.53(0.02-0.65)	12	13	54.5(11/38)	50(10/35)
N+C aa	277	262	239	1954	0.633	0.591	1	0.64(0.01-0.81	12	13	55.5(11/39)	49(10/34)

nu = nucleotide, aa = amino acid, chr = total # characters, Var. = variable characters, PI = parsimony informative characters, Len = tree length, CI = consistency index of most parsimonious tree(s) for given data, RI = retention index, MPT=# most parsimonious trees.

**Figure 4:**
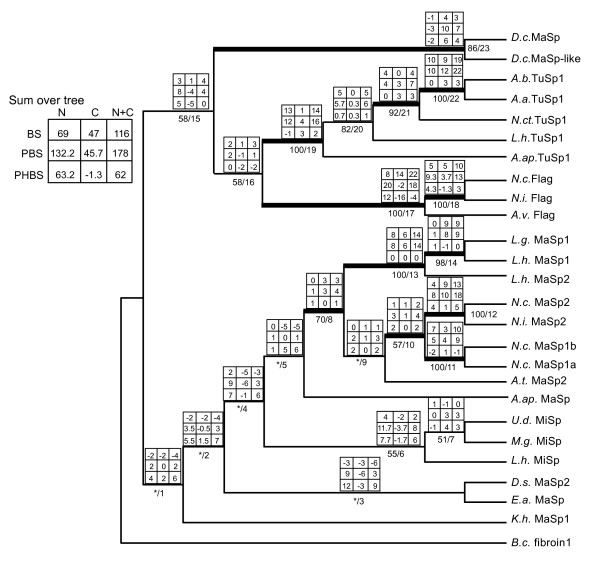
**Combined parsimony analysis of spidroin N- and C-termini showing consensus and conflict among domains**. Matrix above branches shows character support by partition, and summed across tree in top left legend. Matrix columns from left to right: N-terminus, C-terminus, N+C termini; Rows from top to bottom: BS = branch support (decay index), PBS (partitioned branch support, and PHBS (partitioned hidden branch support). Below branches, left of the slash = bootstrap support (* = < 50%), right of the slash = node # referred to in text, thickened branches supported > 70% bootstrap replicates in parsimony nucleotide analysis (Additional file 5). Note that the *K.h*. MaSp1 C-terminus was coded as missing data, and the N-terminus of *D.s*. MaSp2 was concatenated with the *D.s*. MaSp2a C-terminus.

**Figure 5:**
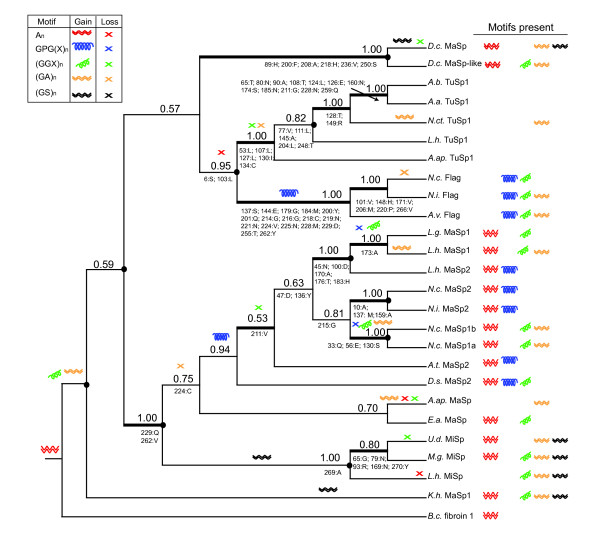
**Bayesian consensus tree from spidroin N- and C-termini reconstructing structural motifs and gene duplications**. 50% majority-rule consensus of post-burnin Bayesian trees from combined spidroin N+C terminal domains. Numbers above branches indicate PP values; thickened branches supported > 0.95 PP in Bayesian nucleotide analysis (Additional file 5b). Circles at nodes show inferred gene duplication events. Shown below branches are terminal domain amino acid synapomorphies by position (N-terminal positions from 1-168; C-terminal positions from 169-277). Gain of motif along a branch indicated as symbol in legend, and loss by an "X" of the same color. There are other equally parsimonious reconstructions for the evolution of (GA)_n _and (GGX)_n _motifs to those shown.

In parsimony and Bayesian nucleotide and amino acid analyses, the combination of N-and C-terminal data increased the number of strongly supported nodes (BT ≥ 70; PP ≥ 0.95) over those in separate N- and C-terminal trees (Figures 3, 4 and 5, Table 2, Additional files 4 and 5). The Bayesian consensus tree for the combined amino acid data was completely resolved and nearly identical to the single most parsimonious tree from that data (Figures 4 and 5) The main difference was in the placement of *D.s*. MaSp2, which in the parsimony tree grouped with *E.a*. MaSp, but was sister to the araneoid MaSp1 and MaSp2 sequences in the Bayesian consensus. Combined amino acid trees included a TuSp1 clade (BT = 100, PP = 1.00), a Flag clade (BT = 100, PP = 1.00), and a MiSp clade (BT = 55, PP = 1.00), but MaSp sequences were paraphyletic (Figures 4 and 5). Species tree reconciliation analyses with every spidroin phylogeny indicated that rooting on the branch leading to *Bothriocyrtum californicum *fibroin 1 minimized costs associated with gene duplications and losses.

Evidence for any strongly supported, but conflicting nodes between N- and C-terminal trees was limited to the TuSp1 clade, which in the Bayesian C-terminal nucleotide analyses included all Flag sequences (PP = 0.98; Additional file 4). By contrast, the N-terminal Bayesian nucleotide tree showed a well-supported monophyletic TuSp1 (PP = 1.00), which was in agreement with the results of all other analyses. Also, *L.h*. TuSp1 grouped with *A.ap*.TuSp1 in the C-terminal amino acid Bayesian tree (PP = 0.98), which conflicts with it being sister to all other TuSp1 sequences in the N-terminal Bayesian amino acid tree (PP = 1.00; Figure 3). However, partition-homogeneity tests did not indicate significant incongruence between the N- and C-terminal characters (nu: P = 0.788; aa: P = 0.330). Comparisons of alternative topologies using the Shimodaira-Hasegawa (SH) test indicated there were significant differences in likelihood between topologies derived from separate N- or C-terminal data analyses (Table 3). But parsimony and Bayesian combined (N+C) data topologies were not significantly different from the best likelihood N- or C-terminal trees from separate data analyses. The sum of N- and C-terminal Partitioned Branch Support (PBS) values for all nodes in the combined parsimony tree (Figure 4) indicated that N-terminal data (PBS sum = 132.2) contributed much more support than the C-terminal data (PBS sum = 45.7). Nearly all N-terminal PBS scores were positive, whereas several C-terminal PBS scores were negative, the largest negative value being -6 (node 4, Figure 4). The Hidden Branch Support (HBS) values indicated that there was hidden support gained by combining the data (HBS sum = 62) and only 3 of 23 nodes indicated some hidden conflict (nodes 11, 16, and 17, Figure 4). Much of the hidden support emerged from the N-terminal data, while hidden conflict was mainly restricted to the C-terminal data (see PHBS values, Figure 4).

**Table 3:** Summary of SH tests comparing alternative phylogenetic hypotheses.

Data	model	Hypothesis^a ^	-ln L Score	-ln L Difference	**Prob**.
N term nu	HKY+I+G	Nterm nu Highest PP	10680.106	BEST	1.0000
		Nterm nu 1 of 1 MPT	10687.329	7.222	0.557
		Cterm nu Highest PP	10744.788	64.681	0.000*
		Cterm nu 1 of 2 MPT	10734.517	54.410	0.000*
		Cterm nu 2 of 2 MPT	10731.541	51.435	0.000*
		N+C term nu Highest PP	10689.373	9.266	0.431
		N+C term nu 1 of MPT	10694.996	14.889	0.247
C term nu	TrN+G	Nterm nu, Highest PP	6812.899	28.163	0.036*
		Nterm nu 1 of 1 MPT	6815.562	30.826	0.016*
		Cterm nu Highest PP	6784.735	BEST	1.0000
		Cterm nu 1 of 2 MPT	6791.424	6.688	0.544
		Cterm nu 2 of 2 MPT	6791.653	6.917	0.574
		N+C term nu Highest PP	6792.463	7.728	0.526
		N+C term nu 1 of MPT	6795.710	10.974	0.333
Nterm aa	WAG+G	Nterm aa Highest PP	5630.28	BEST	1.0000
		Nterm aa 1 of 4 MPTs	5644.44	14.15	0.4050
		Nterm aa 2 of 4 MPTs	5646.50	16.22	0.3670
		Nterm aa 3 of 4 MPTs	5635.67	5.39	0.7000
		Nterm aa 4 of 4 MPTs	5633.38	3.09	0.8200
		Cterm aa Highest PP	5665.89	35.60	0.0330*
		Cterm aa 1 of 8 MPTs	5666.24	35.95	0.0350*
		Cterm aa 2 of 8 MPTs	5668.54	38.25	0.0270*
		Cterm aa 3 of 8 MPTs	5662.15	31.86	0.0550
		Cterm aa 4 of 8 MPTs	5666.87	36.59	0.0360*
		Cterm aa 5 of 8 MPTs	5674.10	43.82	0.0120*
		Cterm aa 6 of 8 MPTs	5669.18	38.89	0.0230*
		Cterm aa 7 of 8 MPTs	5676.45	46.16	0.0070*
		Cterm aa 8 of 8 MPTs	5664.36	34.04	0.0370*
		N+C term aa Highest PP	5640.57	10.29	0.5840
		N+C term aa 1 of 1 MPT	5636.82	6.54	0.7770
Cterm aa	JTT+G	Nterm aa Highest PP	3404.20	38.83	0.0060*
		Nterm aa 1 of 4 MPTs	3415.87	50.50	0.0030 *
		Nterm aa 2 of 4 MPTs	3433.35	67.98	0.0000 *
		Nterm aa 3 of 4 MPTs	3438.46	73.09	0.0000 *
		Nterm aa 4 of 4 MPTs	3421.55	56.18	0.0040 *
		Cterm aa Highest PP	3365.37	BEST	1.0000
		Cterm aa 1 of 8 MPTs	3378.26	12.89	0.3920
		Cterm aa 2 of 8 MPTs	3378.20	12.82	0.3940
		Cterm aa 3 of 8 MPTs	3381.57	16.20	0.2860
		Cterm aa 4 of 8 MPTs	3381.57	16.20	0.2860
		Cterm aa 5 of 8 MPTs	3382.67	17.30	0.2740
		Cterm aa 6 of 8 MPTs	3381.54	16.16	0.2950
		Cterm aa 7 of 8 MPTs	3382.62	17.25	0.2790
		Cterm aa 8 of 8 MPTs	3381.54	16.16	0.2950
		N+C term aa Highest PP	3376.32	10.95	0.4780
		N+C term aa 1 of 1 MPT	3394.02	28.65	0.0310*

a. Highest PP is Bayesian topology with highest posterior probability, * indicates topology statistically different from best topology for a given dataset and model. aa = amino acids, nu = nucleotides.

In the combined analyses, although the N-termini contributed more phylogenetic support, there were more synapomorphic residues in the C-terminal data (43) than in the N-termini (37; Figure 5). Considering the combined analysis results, ancestral reconstruction of structural motifs scored from exemplar repeats (Additional files 6 and 7) showed the unambiguous presence of A_n _motifs at the root of the tree and its loss in three independent lineages (Figure 5). GPGX_n _repeats evolved at least twice to explain their presence in Flag sequences and in the clade containing MaSp2 sequences. (GS)_n _motifs independently arose in the MiSp lineage and in *K.h*. MaSp1. Alternative reconstructions were possible for (GA)_n _and (GGX)_n _evolution, as the presence or absence of these motifs at the root of the tree was equivocal (Figure 5).

## Discussion

### Conservation and diversity of spidroin N-termini

Despite years of intense research aimed at discovering the molecular basis for spider silk mechanics, determination of full-length spidroin sequences has rarely been achieved [11,12]. While many partial spidroin fragments containing C-termini have been characterized from divergent spider species, very few N-terminal sequences of these proteins are known and all but one were from araneoid species [11,12,18-21,34]. The limited number of the non-repetitive N-terminal domain sequences has restricted generalizations regarding its distribution across spidroins, as well its variability and potential functional significance. In this study we substantially expanded the number and diversity of spidroin N-terminal domain sequences by employing an EST approach, where > 1,900 silk gland cDNAs were sequenced at random. Using this method, we discovered that spidroins from the divergent spider lineages Mygalomorphae, Haplogynae, Agelenoidea, and Deinopoidea are also characterized by the presence of a non-repetitive N-terminal domain with high sequence similarity to those reported from distantly related araneoid spiders [11,12,18-20,34] and the pisaurid *Euprosthenops australis *[21].

The finding of an N-terminal domain in a mygalomorph spidroin is significant because it indicates that this molecular feature has been conserved for at least 240 million years of spider silk production, the minimal age that fossil evidence dates the divergence of mygalomorphs (tarantulas and their kin) and araneomorphs ("true spiders") from a common ancestor [56]. Molecular dating with multiple fossil calibration points estimates an even older divergence, as early as ~390 million years ago [33]. The conserved N-terminal domain may be a universal feature of spidroin protein architecture that has persisted since the spinning of the first spider silk, estimated to have occurred in the Devonian.

Our analyses show that spidroin N-termini are relatively conserved at multiple levels of molecular organization. In addition to sequence similarity, the different N-termini are similar in length (~151-162 amino acids) and share a common translation initiation site. After the first methionine, two other residues are identical across all spidroins (position 70 = D and position 140 = G), while their corresponding C-terminal domains do not have a single residue that is 100% conserved. The shared features of spidroin N-termini also include predicted signal peptides, hydropathy profiles characterized by similarly alternating hydrophobic and hydrophilic regions, and secondary structural predictions consisting of 4-6 alpha-helices. While previous studies noted some of these characteristics [20,21], our work substantially broadens their distribution across very distantly related species and spidroin paralogs that compose mechanically dissimilar silks.

Previous mass spectrometry (MS) work confirmed the presence of the TuSp1 N-terminal domain in egg-case silk fibers [18]. The *Latrodectus hesperus *MiSp N-terminus we report here contains the sequences (VWDSTATAEAFIGSFNS and MDDISSISDTIISAIER) that exactly match mass spectrometry peptide sequences collected from *L. hesperus *minor ampullate silk by LaMattina et al. [57] (peptide mass 2081.0 and 2434.1), excepting that all "I" residues were reported as "L" (I and L are difficult to distinguish with MS). These data indicate that the MiSp N-terminal sequence (beyond the signal cleavage site) is also present in minor ampullate silk fibers and given the conserved characteristics, further reinforces that this domain contributes to silk production beyond secretory signaling.

Though relatively similar at the structural level, the new N-terminal sequences we report reveal far more sequence diversity than previously known. Our work confirms that this domain is the most conserved region across spidroin paralogs, although it exhibits only slightly greater pairwise identity than do its corresponding C-termini. This additional N-terminal sequence diversity is intriguing because it may relate to differential mechanisms of fiber formation among functionally distinct silks. Fiber assembly processes are likely to vary across gland types because of the differences in the repetitive structural motifs of their constituent spidroins and the length of time they are stored in glands (e.g., major ampullate silk is used daily while tubuliform silk is used only a few times in a spider's lifetime). Examples of paralog-specific features are the two identically positioned cysteine residues that have evolved in araneoid and non-araneoid TuSp1 N-termini (positions 52 and 134, Additional file 1), suggesting their involvement in a biochemical mechanism particular to the assembly of spider egg-case silk fibers. These cysteines may participate in intra- or inter-molecular disulfide bridges, much like the conserved cysteines in lepidopteran heavy-chain fibroin [18,58].

### Phylogenetic utility of spidroin N-termini

It is generally not controversial to combine sequence data from different regions of the same gene or protein for phylogenetic reconstruction. However, a number of features specific to spidroin genes suggest that their evolutionary dynamics may create phylogenetic conflict between the two termini. Recent work has demonstrated that there are multiple genomic copies of the *MaSp1 *dragline silk gene [8,21,34]. Detailed evolutionary analyses of *Latrodectus MaSp1 *and *MaSp2 *genes showed their encoded N-and C-termini do not form mutually exclusive clades, despite having markedly different repetitive region sequences (e.g., GPGX_n _motifs are abundant in MaSp2 but absent in MaSp1; [8]). Relationships among the N-termini of these sequences also conflicted with those from their C-termini, which was attributed to intergenic recombination between different *MaSp1 *copies and also between *MaSp1 *and *MaSp2 *[8]. Such recombination would introduce conflict in combined phylogenetic analyses. Ayoub and Hayashi [8] hypothesized that an alternative explanation for the unexpected groupings of MaSp1 and MaSp2 termini could be convergent evolution within a genome to facilitate co-expression and/or co-assembly in major ampullate glands. Convergent evolution of N- and/or C-termini would obscure their phylogenetic relationships in either separate or combined analyses.

Regardless of the potential for recombination and convergence, we primarily observe congruence among the well-supported nodes in trees separately constructed from N- and C-termini and a partition homogeneity test did not find strong evidence of character conflict between the two datasets. While there were significant differences in the likelihood scores of topologies produced by the N- and C-termini, neither N- nor C-terminal topologies were significantly different from the combined topologies. Our analyses did show repeated grouping of araneoid MaSp1 with MaSp2 sequences, but these relationships are mirrored in both N- and C-terminal trees where they were strongly supported. Some disagreement was found between N- and C-termini within the TuSp1 clade, suggesting the need for additional sequences to determine if this conflict persists or is a sampling artifact. Despite our best efforts to link N- and C-termini from the same protein, there is the possibility that in some cases the concatenated termini do not represent two ends of the same molecule, but instead are from different paralogs, introducing another potential source of phylogenetic conflict.

The combination of N- and C-termini produced improved phylogenies over separate analyses, based on the criteria of containing more strongly supported branches, increased branch support values, and being more robust to different methods of phylogenetic inference (i.e., the parsimony tree and Bayesian consensus were highly congruent). Combination of the data also revealed a much higher level of hidden branch support relative to hidden conflict [53], consistent with an overall increase in phylogenetic signal. However, the contribution of the two termini to combined analyses was imbalanced, as indicated by the partitioned branch support and hidden partitioned branch support values that showed N-termini provided greater support than C-termini. This result suggests that N-termini are more informative than C-termini for understanding spidroin relationships, perhaps because of their greater length and sequence conservation. Future work will focus on characterizing the presently unknown N-terminal domains of spidroins composing prey-wrapping silk (AcSp1; [59]) and cementing silk (PySp1 [60]) to further clarify spider silk diversification.

### Evolution of spider silks

Although all spiders make silk, perhaps the greatest complexity of silk production is displayed by araneoid orb-weavers, which possess seven distinct gland types that manufacture different silks with diverse functional applications. Molecular characterizations of araneoid silks has established that six of these glands (major ampullate, minor ampullate, flagelliform, aciniform, tubuliform and pyriform glands) each express unique combinations of spidroin paralogs [9,19,29,59-61]. These paralogs encode proteins with varying proportions of structural motifs that underlie the signature mechanical properties of each fiber type. Outside of araneoids, there is tremendous variation in the number and types of silk glands, as well as the set of spidroin paralogs found across species. The evolution of this striking diversity can be investigated by jointly considering: 1) the phylogenetic relationships of spiders; 2) the distribution of silk glands among these lineages; and 3) the relationships of spidroins expressed by these glands and their sequence features.

In contrast to araneoids (part of the suborder Araneomorphae), spiders in the suborder Mygalomorphae (tarantulas and their kin) possess many primitive features of silk production, including homogeneous, acinous-shaped silk glands and uniform fiber types [62]. Consistent with their lesser glandular diversification, mygalomorphs also express fewer spidroin paralogs than araneomorphs and these paralogs are also relatively similar in sequence [7]. The glandular affiliation hypothesis of Hayashi and Lewis [19] proposed that spidroins evolved in association with the glands where they are primarily expressed, predicting a phylogenetic correlation between araneomorph gland type and spidroin paralogs. Our combined N- and C-terminal spidroin amino acid trees generally support this expectation: TuSp1, Flag and MiSp, expressed in tubuliform, flagelliform and minor ampullate silk glands, respectively, each form mutually exclusive clades. However, spidroins characterized from the major ampullate glands of the haplogyne species *Kukulkania *(*K.h*. MaSp1) and *Diguetia *(*D.c*. MaSp and *D.c*. MaSp-like) did not group with MaSp sequences from Entelegynae species. While all araneomorph spiders possess major ampullate glands, tubuliform glands are restricted to the Entelegynae clade, and flagelliform (and homologous pseudoflagelliform) glands subsequently evolved in the common ancestor of Araneoidea and Deinopoidea. Accordingly, tubuliform and flagelliform glands (and their expressed spidroins) may have originated as duplicates of major ampullate glands. There has been apparent duplication and loss of major ampullate glands among araneomorph spider lineages [63], such that the major ampullate glands of Haplogynae and Entelegynae spiders also may not be strictly homologous (identity of structures through inheritance from a common ancestor). Instead, some major ampullate glands may be serially homologous to each other (similarity of structures due to common developmental mechanisms). The non-monophyly of MaSp sequences may also reflect our greater sampling of major ampullate gland cDNAs from a wider range of species in distantly related spider families, as compared to our more limited sampling of other silk gland types in non-orbicularian species. Thus the grouping of TuSp1, Flag and MiSp into monophyletic clades could break down with further taxonomic sampling, suggesting that future work should substantially increase sampling of silk sequence types from a more diverse and numerous set of spider taxa.

The disjunct relationships of major ampullate spidroin termini are not entirely surprising given the diversity of their repetitive sequences. Major ampullate spidroins from orbicularian (araneoid + deinopoid) species, are largely characterized by iterations of A_n _in combination with either tandem arrayed GGX in MaSp1 or GPGX_n _in MaSp2. The repetitive sequence of major ampullate spidroins from the haplogyne *Kukulkania *[55] and *Diguetia *(this study) are distinct from each other and from orbicularian MaSps (see Additional file 6). For instance, *D.c*. MaSps, characterized from the ampullate shaped glands of *Diguetia *contain A_n_, but are unusual in also containing strings of glutamine (Q_n_). *K.h*. MaSp1, described from the major ampullate glands of *Kukulkania *[53], contains many iterations of (GA)_n _and (GS)_n_, and much less A_n _than in orbicularian MaSps and no Q_n _like the *D.c*. MaSps. Swanson et al. [2] found that major ampullate silk fibers from divergent spider species exhibit substantial variability in their mechanical properties, which may correlate with the phylogenetic distribution of structural motifs we observe in MaSp repetitive sequences (Figure 5). Our results indicate that the structural module A_n_, primarily associated with the high tensile strengths of major ampullate silk, was present in ancestral spidroins but was subsequently lost in some paralogs or expanded in others.

The relatively close relationship between the terminal domains of the egg-case silk protein TuSp1 and the orb-web capture spiral silk protein Flag is especially surprising given their dissimilar functions and repetitive sequence properties. A correlation between spidroin phylogeny and silk ecological use might predict a close relationship between Flag and other silk proteins used in orb-webs, such as the temporary scaffolding protein MiSp. Flag may alternatively be expected to share recent ancestry with orbicularian MaSp2 proteins, because both contain numerous iterations of the proline containing GPGX_n _structural module that forms elastic nano-springs [15]. Our phylogenetic hypothesis instead suggests more radical shifts in silk use subsequent to spidroin gene duplication, as well as convergent evolution of GPGX_n _modules that impart fibers with greater extensibility.

## Conclusions

The presence of a similar, non-repetitive N-terminal domain in spidroin proteins across divergent spider lineages supports its participation in a general mechanism of spider silk production. Sequence conservation of these N-termini makes them an unequalled resource for reconstructing spidroin phylogeny. The improved understanding of spidroin relationships we provide using both N- and C-terminal domains shows that there is considerable evolutionary flexibility throughout the spider silk system, from the level of gene sequence motif to paralog number and silk gland expression pattern. This dynamic, labile nature of silk evolution is in stark contrast to the incredible homogeneity of repeats within some spidroins (e.g., 100% identity in consecutive 1026 bp repeats; [7]) and the consistently high-performing mechanical properties of silk fibers (e.g., dragline silks, [2]). Given the elevated rate of sequence rearrangement and turnover in the repetitive region, the spidroin N- and C-terminal domains are not only important for the biochemistry of silk fiber production, but also serve as signposts for retracing the history of the ancient and functionally diverse spider silks.

## Authors' contributions

JEG and CYH conceived this study. JEG and NAA collected the data. JEG and CYH analyzed the data and wrote the manuscript. All authors read and approved the final manuscript.

## Supplementary Material

Additional file 1 **N-terminal alignment, top line shows residues in 50% or more sequences, boxed in region indicates most probable signal peptide region as predicted in SignalP**. Sequence names abbreviated as in Table 1. Missing data indicated by X and alignment gaps by dashes. Click here for file

Additional file 2 **Superimposed Kyte-Doolittle plots for N-terminal alignment indicating hydropathy**. X-axis indicates residue position along alignment, Y-axis shows hydropathy score, where values above 0 indicate hydrophobicity and values below zero indicates hydrophilicity. Each line represents a different sequence. Breaks within lines correspond to gapped regions in sequence alignment. Click here for file

Additional file 3 **Secondary structure predictions for representative spidroin N-terminal sequences**. A-G. Distribution of three predicted structures 1: Alpha-helices (long, blue lines), 2. Extended strand (medium height, red lines) and 3. random coils (short, purple lines) predicted with GOR IV in varied spidroins, sequence names abbreviated as in Table 1; A: *B.c*. fibroin1, B: *K.h*. MaSp1, C: *A.ap*. TuSp1, D: *D.c*. MaSp, E: *L.h*. MiSp, F: *D.s*. MaSp2, G: *N.i*. Flag. Sequences from first residue following predicted signal peptide. H. Table showing percentage of three structures in each spidroin. Click here for file

Additional file 4 **Spidroin terminal phylogenies based on nucleotides encoding protein in Additional file **1.; A. N-terminal parsimony tree, B. N-terminal Bayesian consensus tree; C: C-terminal parsimony strict consensus tree; D. C-terminal Bayesian tree; A, C Numbers above nodes are bootstrap values, numbers below nodes are decay indices; B, D numbers above nodes are clade posterior probability values. Click here for file

Additional file 5 **Combined spidroin N+C terminal nucleotide analyses**. A. 1 MPT; Above node, bootstrap support, below node, branch support (decay index). B. 50% majority-rule consensus of post-burnin Bayesian trees from combined N+C nucleotides, numbers indicate PP values. Click here for file

Additional file 6 **Exemplar repeats used in motif coding analyses**. Each exemplar represents a repeat taken from the complete sequence (e.g., *N.c*. MaSp1a), minor variants of these repeats are tandem iterated throughout the complete sequence. Click here for file

Additional file 7 **Presence or absence of structural motifs in spidroin exemplar repeats**. 0 = absent, 1 = present. Click here for file
