# Supplementary material for: Untangling spider silk evolution with spidroin terminal domains
Source: BMC Evol Biol. 2010 Aug 9;10:243. doi: 10.1186/1471-2148-10-243 (PMC2928236; doi:10.1186/1471-2148-10-243)
Supplement: Additional file 1 — N-terminal alignment, top line shows residues in 50% or more sequences, boxed in region indicates most probable signal peptide region as predicted in SignalP. Sequence names abbreviated as in Table 1. Missing data indicated by X and alignment gaps by dashes. [file 1471-2148-10-243-S1.PDF]

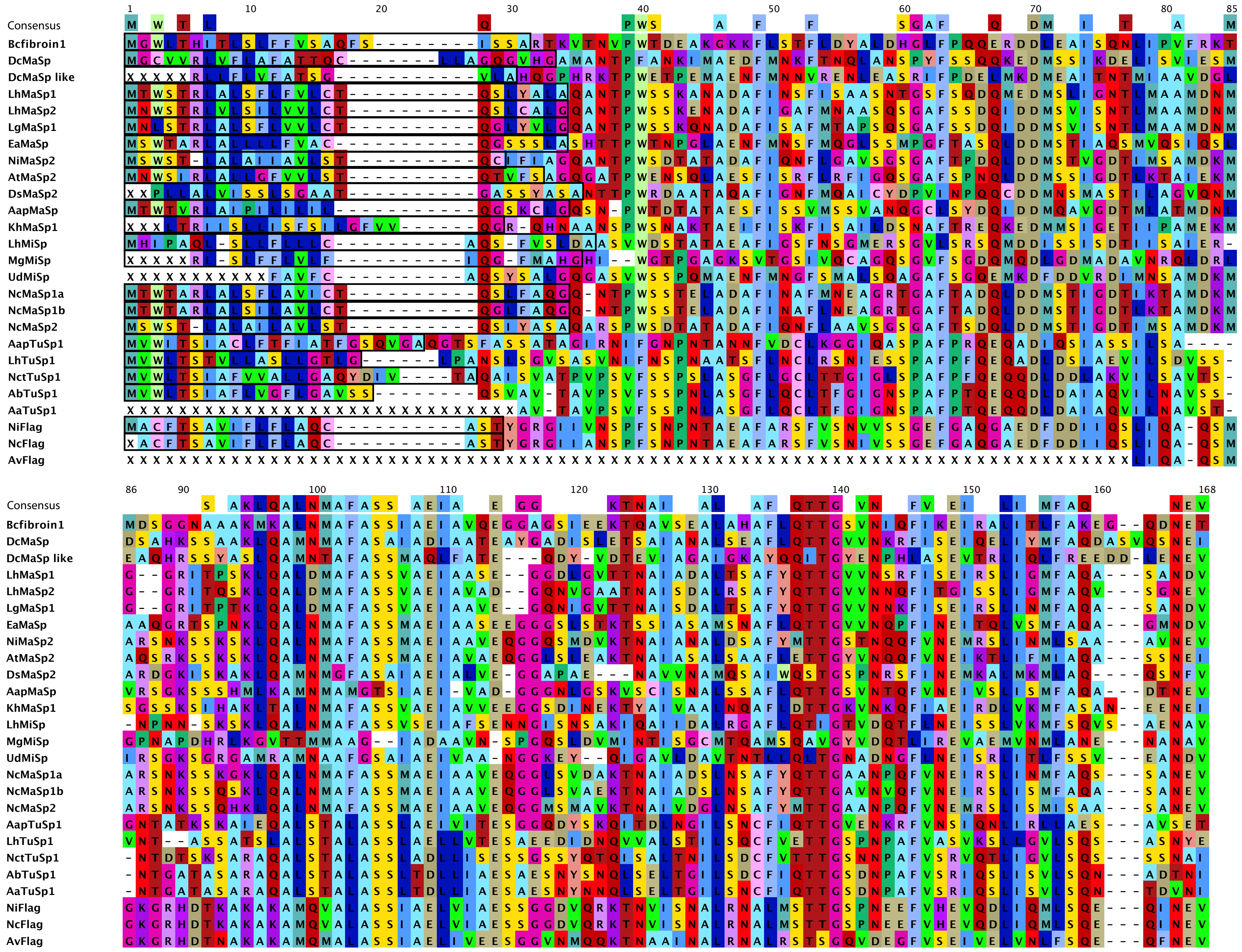

Additional file 1. N-terminal alignment, top line shows residues in 50% or more sequences, boxed in region indicates most probable signal peptide region as predicted in SignalP. Sequence names abbreviated as in Table 1. Missing data indicated by X and aligment gaps by dashes.
