# Supplementary material for: Untangling spider silk evolution with spidroin terminal domains
Source: BMC Evol Biol. 2010 Aug 9;10:243. doi: 10.1186/1471-2148-10-243 (PMC2928236; doi:10.1186/1471-2148-10-243)
Supplement: Additional file 2 — Superimposed Kyte-Doolittle plots for N-terminal alignment indicating hydropathy. X-axis indicates residue position along alignment, Y-axis shows hydropathy score, where values above 0 indicate hydrophobicity and values below zero indicates hydrophilicity. Each line represents a different sequence. Breaks within lines correspond to gapped regions in sequence alignment. [file 1471-2148-10-243-S2.PDF]

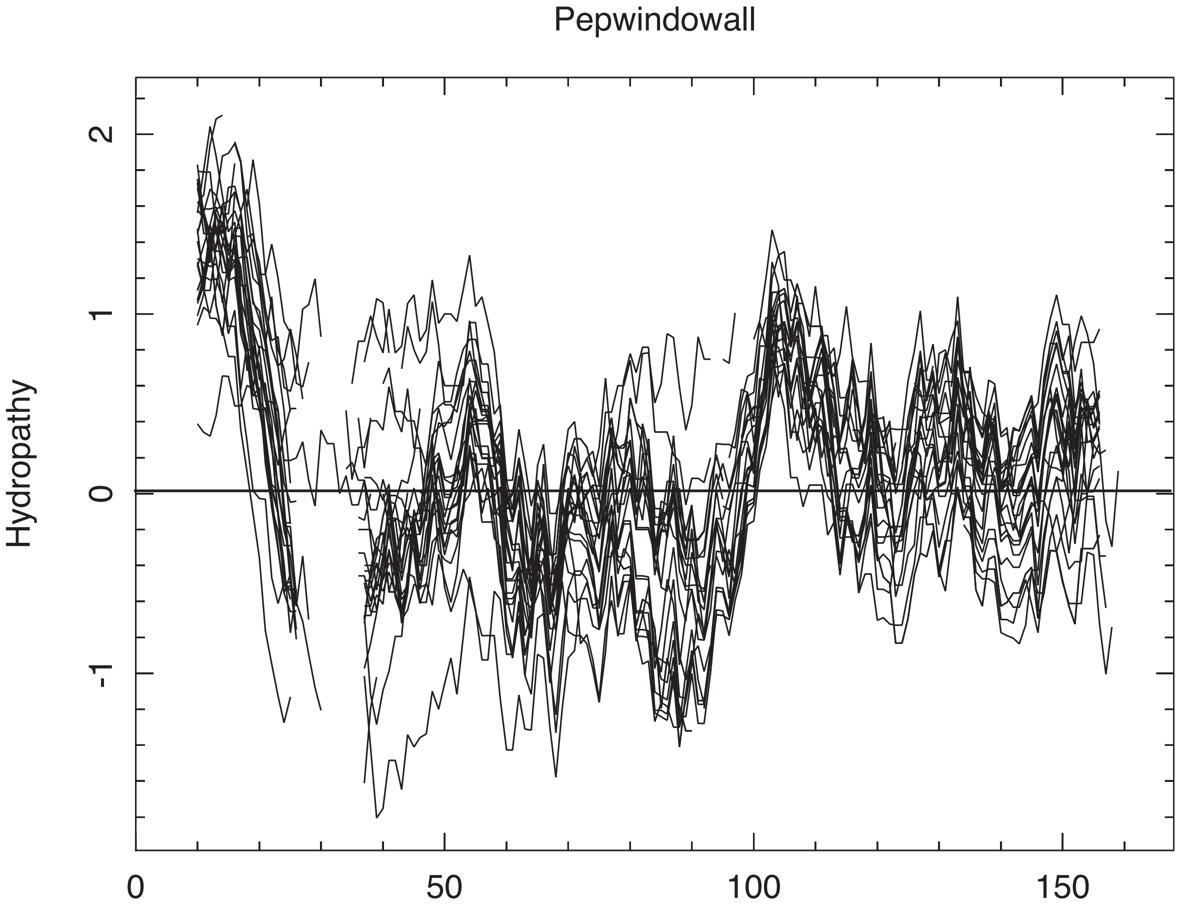

Additional file 2. Superimposed Kyte-Doolittle plots for N-terminal alignment indicating hydropathy. X-axis indicates residue position along alignment, Y-axis shows hydropathy score, where values above 0 indicate hydrophobicity and values below zero indicates hydrophilicity. Each line represents a different sequence. Line gaps correspond to gapped regions in sequence alignment.
