# Supplementary material for: Untangling spider silk evolution with spidroin terminal domains
Source: BMC Evol Biol. 2010 Aug 9;10:243. doi: 10.1186/1471-2148-10-243 (PMC2928236; doi:10.1186/1471-2148-10-243)
Supplement: Additional file 3 — Secondary structure predictions for representative spidroin N-terminal sequences. A-G. Distribution of three predicted structures 1: Alpha-helices (long, blue lines), 2. Extended strand (medium height, red lines) and 3. random coils (short, purple lines) predicted with GOR IV in varied spidroins, sequence names abbreviated as in Table 1; A: B.c. fibroin1, B: K.h. MaSp1, C: A.ap. TuSp1, D: D.c. MaSp, E: L.h. MiSp, F: D.s. MaSp2, G: N.i. Flag. Sequences from first residue following predicted signal peptide. H. Table showing percentage of three structures in each spidroin. [file 1471-2148-10-243-S3.PDF]

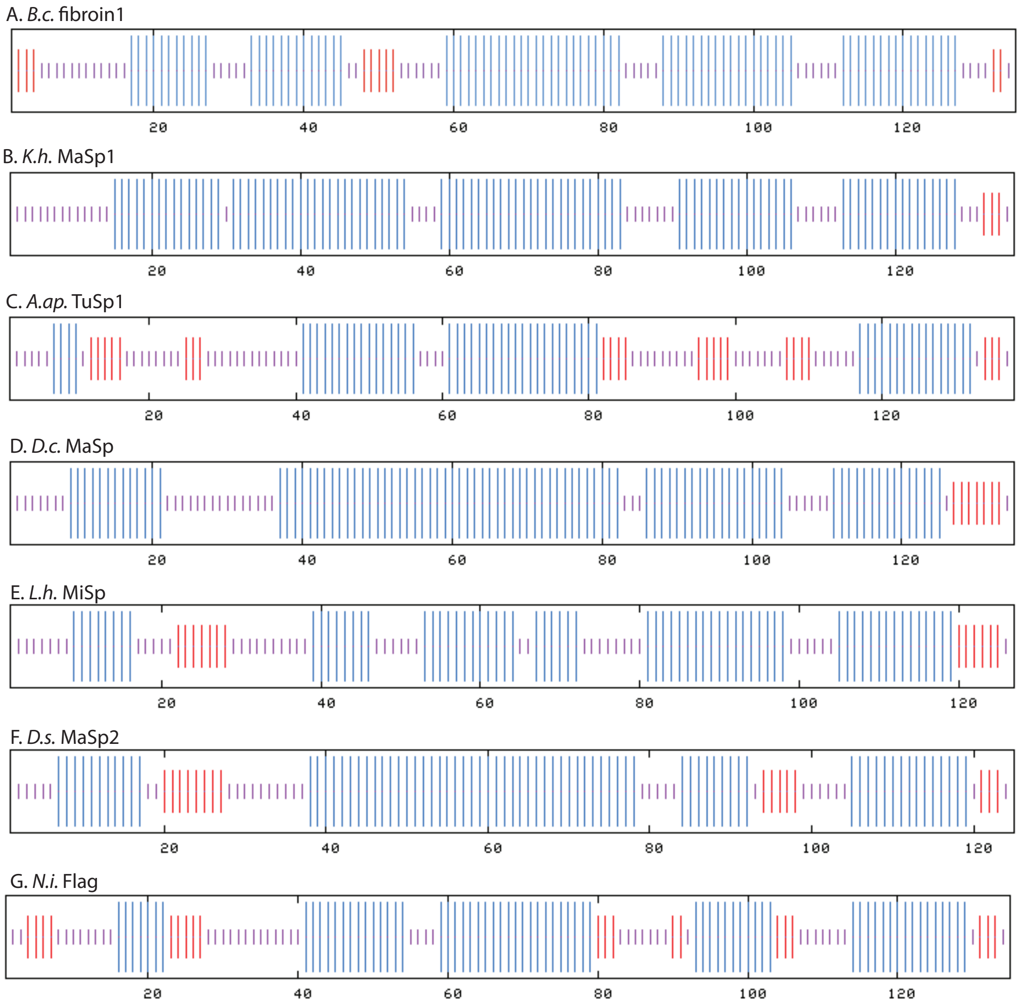

H.

	Alpha-Helix	Extended Strand	Random Coil
B.c. fibroin1	60.74	7.40	31.85
K.h. MaSp1	70.59	2.21	27.21
A.ap. TuSp1	41.30	17.90	41.30
D.c. MaSp	68.89	5.19	25.93
L.h. MiSp	52.76	10.24	37.01
D.s. MaSp2	60.80	12.80	26.40
N.i. Flag	51.11	14.81	34.07

Additional file 3. Secondary structure predictions for representative spidroin N-terminal sequences. A-G. Distribution of three predicted structures 1: Alpha-helices (long blue lines), 2. Extended strand (medium height red lines) and 3. random coils (short, purple lines) predicted with GOR IV in varied spidroins, sequence names abbreviated as in Table 1; A: *B.c.* fibroin1, B: *K.h.* MaSp1, C: *A.ap.* TuSp1, D: *D.c.* MaSp, E: *L.h.* MiSp, F: *D.s.* MaSp2, G: *N.i.* Flag. Sequences from 1st residue following predicted signal peptide. H. Table showing percentage of three structures in each spidroin.
