# Supplementary material for: Untangling spider silk evolution with spidroin terminal domains
Source: BMC Evol Biol. 2010 Aug 9;10:243. doi: 10.1186/1471-2148-10-243 (PMC2928236; doi:10.1186/1471-2148-10-243)
Supplement: Additional file 4 — Spidroin terminal phylogenies based on nucleotides encoding protein in Additional file 1.; A. N-terminal parsimony tree, B. N-terminal Bayesian consensus tree; C: C-terminal parsimony strict consensus tree; D. C-terminal Bayesian tree; A, C Numbers above nodes are bootstrap values, numbers below nodes are decay indices; B, D numbers above nodes are clade posterior probability values. [file 1471-2148-10-243-S4.PDF]

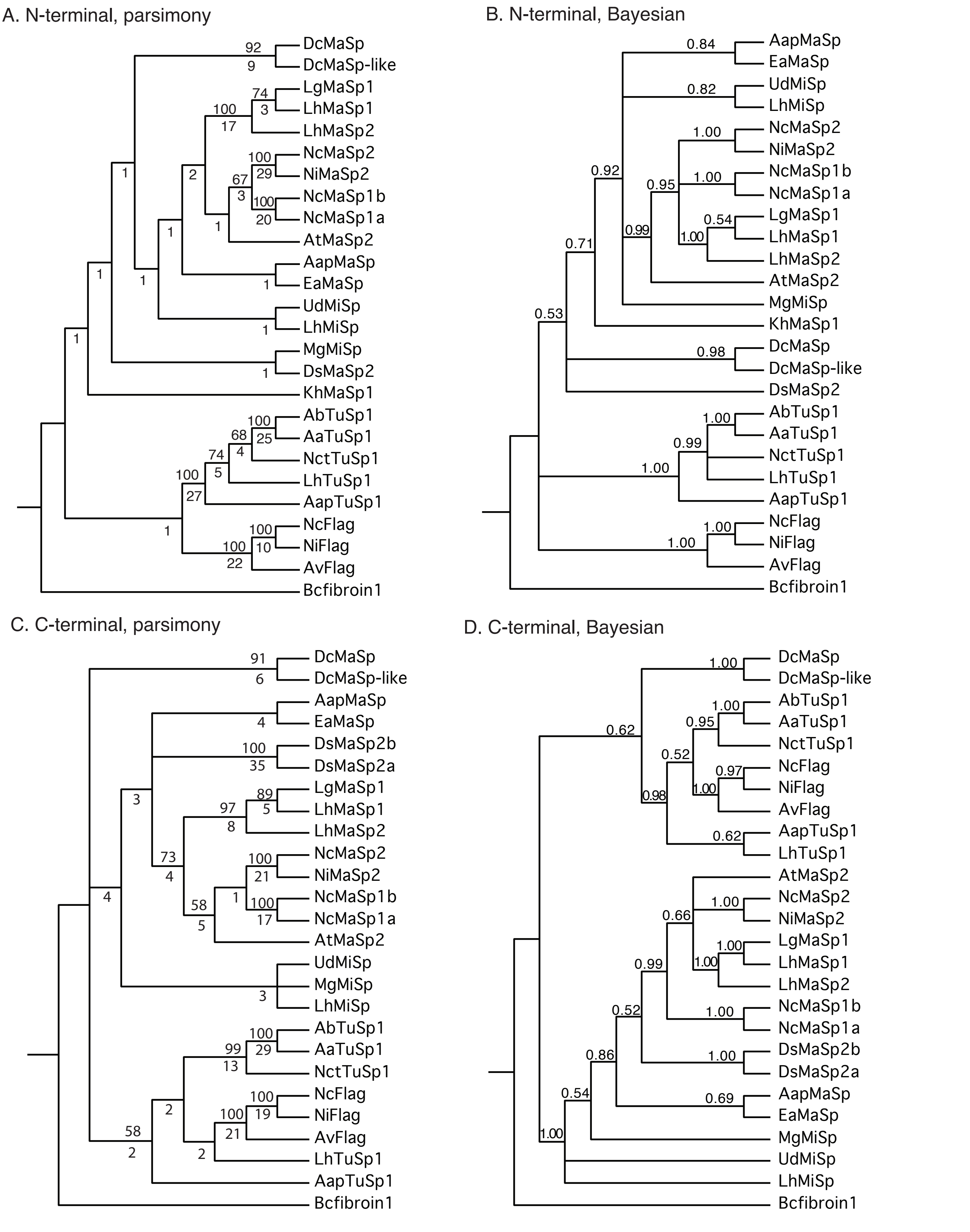

Additional file 4. Spidroin terminal phylogenies based on nucleotides encoding protein in Fig. S1.; A. N-terminal parsimony tree, B. N-terminal Bayesian consensus tree; C: C-terminal parsimony strict consensus tree; D. C-terminal Bayesian tree; A,C Numbers above nodes are bootstrap values, numbers below nodes are decay indices; B,D numbers above nodes are clade posterior probability values.
