# Supplementary material for: Untangling spider silk evolution with spidroin terminal domains
Source: BMC Evol Biol. 2010 Aug 9;10:243. doi: 10.1186/1471-2148-10-243 (PMC2928236; doi:10.1186/1471-2148-10-243)
Supplement: Additional file 5 — Combined spidroin N+C terminal nucleotide analyses. A. 1 MPT; Above node, bootstrap support, below node, branch support (decay index). B. 50% majority-rule consensus of post-burnin Bayesian trees from combined N+C nucleotides, numbers indicate PP values. [file 1471-2148-10-243-S5.PDF]

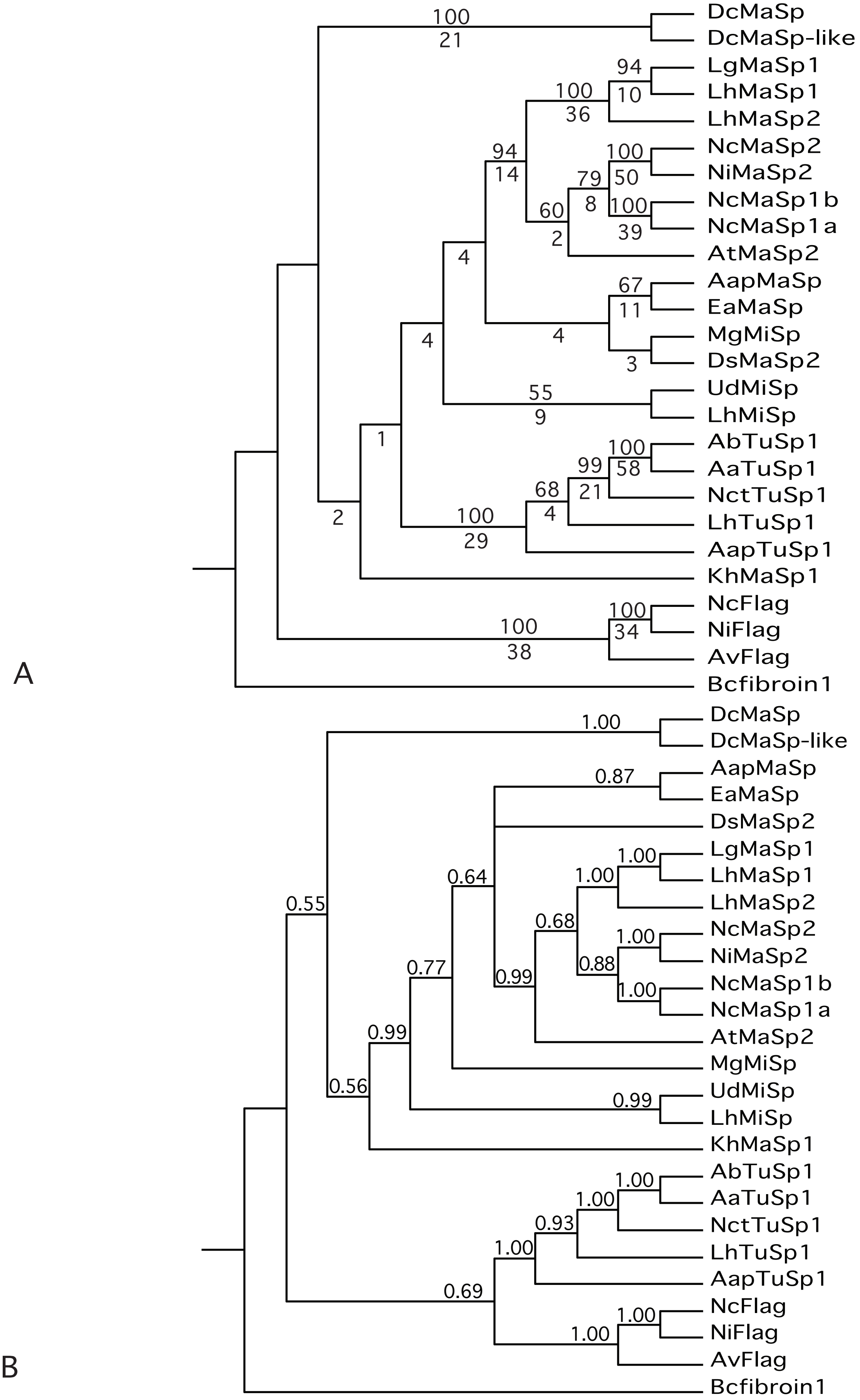

Additional file 5. Combined spidroin N+C terminal nucleotide analyses. A. 1 MPT; Above node, bootstrap support, below node, branch support (decay index). B. 50% majority-rule consenus of post-burnin Bayesian trees from combined N+C terminal nucleotides, numbers indicate PP values.
