# Supplementary material for: Untangling spider silk evolution with spidroin terminal domains
Source: BMC Evol Biol. 2010 Aug 9;10:243. doi: 10.1186/1471-2148-10-243 (PMC2928236; doi:10.1186/1471-2148-10-243)
Supplement: Additional file 6 — Exemplar repeats used in motif coding analyses. Each exemplar represents a repeat taken from the complete sequence (e.g., N.c. MaSp1a), minor variants of these repeats are tandem iterated throughout the complete sequence. [file 1471-2148-10-243-S6.PDF]

Additional file 6: Exemplar repeats used in motif coding analyses.

Each exemplar represents a repeat taken from the complete sequence (e.g., *N.c.* MaSp1a), minor variants of these repeats are tandem iterated throughout the complete sequence.

1) *N.c.* MaSp1a
GGAGQGGYGGLGSQGAGRGAGAAAAAA

2) *N.c.* MaSp1b
GGAGQGGLGGQGAGQGAGAAAAAA

3) *N.c.* MaSp2
GPGQQGPGGYGPGQQGPGGYGPGQQGPSGPGSAAAAAAAAA

4) *L.h.* MaSp1
GGAGQGGQGGYGQGGYGQGGAGQGGAGAAAAAAAA

5) *L.h.* MaSp2
GGAGPGRQQAYGPGGSGAAAAAAA

6) *L.g.* MaSp1
GGAGQGGYLQRGSGQGAAAAAAA

7) *N.i.* MaSp2
GRGPGGYGPGQQGPGGPGAAAAAA

8) *A.t.* MaSp2
GPGYGPGAGQQGPGSQGPGSGGQQGPGGQGPYGPSAAAAAAAA

9) *E.a.* MaSp1
GQGGYGGQGQGGYGQAGGAAAAAAAAAAAAAAAA

10) *D.c.* MaSp
QQQQQQAAAAAAAAAAAAAAGSGQGASFGVTQQFGAPSGAASSAAAAAAAAAAAAAGSG
AGQEAGTGAGAAAAAAAAAGAAGSGAGQGAGSGAGAAAAAAAASAAGAGQGAGSGSGAG
AAAAAAAAAAAA

11) *D.c.* MaSp-like
LFLSSGDFGRGGAGAGAGAAAASAAAASSAAAGARGGSGFGVGTGGFGRGGAGDGASAA
AASAAAASAAAAGAGGD

12) *D.s.* MaSp2
GPGGYGGQGTGQGGQQGPGQGRQGPSAAAAAAAAAAASAA

13) *K.h.* MaSp1
GAGFGSGAGAGSGAGAGYGAGRAGGRGRGGRGGEAFSASSASSAVVFESAGPGEEAGSS
GGGASAAASAAAAAGAGSGRRGPGGARSRGGAGAGAGAGSGVGGYGSGSGAGAGAGAGA

GAGGEGGFGEGQGYGAGAGAGFGSGAGAGAGAGSGAGAGEGVGSGAGAGGAGFGVGAGA
GAGA

14) *A.ap.* MaSp
NYGYGPGVGAGSGAGAGSGSGVGAGSGFGAGAGSGTGLGRGAGTGAGAGSGAGSGAGAG
SGSGAGAGSGSGAGAGRGAGLGGFGSGLGAGAGTGAGAGSGSGAGSGAGAGAGSGFGAG
AGRGAGSGGTGSWLRSGVGTGSSASAGSAGSGAGVGSGAGPGPAYGYAAYYAA

15) *L.h.* TuSp1
SSSTSTTTTTSQAASQAASQSASSSSSAASQSAFSQASSSALASSSSFSSAFSSASSAS
AVGQVGYQIGLNAAQTLGISNAPALADAVSQAVRTVGVGASPFQYANAVSNAFGQLLGG
QGILTQENAAGLASSVSSAISSAASSVAAQAASAAQSSAFAQSQAAAQAFSQAASRSAS
QSAAQAG

16)*A.a.* TuSp1
SSSTTTTTSTSGSQAASQSASSSASQASASSFAQASSASLAASSSFSSAFSSANTLSAL
GNVAYQLGFNVANTLGLGNTAGLGAALSQAVSSVGVGASSATYANAVSNAVGQFLAGQG
ILNGANAASLASSFASALSASAASVASSSAAQSATQSQAAASAFSRAASQSASQSAARS
GAQ

17) *A.ap.* TuSp1
SGAQAASQAASQTSSSSWAAASASTFSQSSASSLASSSAFSSAFSSASSASAVGSLCYQ
LALQTANSLGIQNAASLASAVSQAVSAVGVRASSYAYASAISNTVGQFLISQGLLSQSN
ASALASSFASAFAASAASASASASSSSYAQSSAAAQSQSAASAFSRAASAAASQASSQS
ASQAGSYGRTTTTSTSE

18)*N.ct.* TuSp1
SSSTTTTTSAAASQAASQSASSSSSSAFAQAASSSLATSSAISRAFASVSSASAASSLA
YTIGLSAARSLGIASDTALAGALAQAVAGVGAGASASTYANVIARAAGQFLATQGVLDA
GNASALAGSFARALSASAESQSFAQSQAFQQASAFQQAASQSAGQSASRAGST

19) *A.b.* TuSp1
SSSTTTTTSTSGSQAASQSASSAAAQASASSFAQASSASLAASSAFSSAFSSANSLSAL
GNVGYQIGFNVANTLGIGNAPGLGNALSQAVSSVGVGASSSAYANAVSNAVGQFLAGQG
VLNAGNAGSSLASTFANALSASAASVASSAAAQSAFQSQAAASAFSRAASQSTSQSAAR
SGAQ

20) *B.c.* fibroin 1
SSSSTSTSTTTTSSAAAAASSARSSSAAAASAAAFSSLSRALIYRLQQNQDFIYTFNSI
ETSDAARAITYSSALAAANAMGAGSSASQAVAFAAAKAAEGVPIRSSSYAYAEAITNAI
TPHFLALHLVNSANVDAFASDFTSSFASATASAASASAAASSAASAAAAATA

21) *L.h.* MiSp
GAGGYGQGAGGYGRGQGASAGAGAGAGAGGYGQGAGAGAAAGAAASAGAGGYGQGAGGY
GRGTGAGAGGYGQGTGAGAAAGAGASAGARGYGQRAGGYGQGQGAGAAAGAAAGAGRYG
QGSGSASVSTGGYGQSQVARASSSSAVGTSSSVSTS

22) *M.g.* MiSp

GYGAGAGRGYGAGAGAGAGAVAASGAGAGAGYGAGAGAGAGAGYGAGAGRGYGAGAGAG
AGSGAASGAGAGAGYGAGAGAGAGYGAGAGSGYGTGAGAGAGAAAAGGAGAGAGYGAGA
GRGYGAGAGAGAASGAGAGAGAGAASGAGAGSGYGAGAAAAGGAGAGAGGGYGAGAGRG
YGAGAGAGAGAGSGSGSAAGYGQ

23) *U.d.* MiSp
GAGYGTGAGTGASAGAAASGAGAGYGGQAGYGQGAGASARAAGSGYGAGAGAAAAAGSG
YGAGAGAGAGSGYGAGAAAGSGYGAGAGAGAGSGYGAGAGAGSGYGAGAGAGAGSGYVA
GAGAGAGAGSGYGAGAGAGAGSSYSAGAGAGAGSGYGAGSSASAGSAVSTQTVSSSATT
SSQSAAAAT

24) *A.v.* Flag
GAPGGGPGGAGPGGAGVGPGGAPGAPGGPGGPGGPGGAYGPGAGGPSGPGGAGPGGVGP
YGPGGVGPGGVGPGGAGVGPGGAPGAPGGPGGPGGPGGPGGVGGPLGAGAGGVGPGGAG
AYGPGGAGAYGPGGVGLGGAGAYGPGGAGPGGAGPHGPGGPGGAGPGGEGPVTVDVEVS
VG

25) *N.c.* Flag
GAGGSGPGGAGPGGVGPGGSGPGGVGPGGSGPGGVGPGGSGPGGVGPGGAGGPYGPGGS
GPGGAGGAGGPGGAYGPGGSYGPGGSGGPGGAGGPYGPGGEGPGGAGGPYGPGGAGGPY
GPGGAGGPYGPGGEGGPYGPGGSYGPGGAGGPYGPGGPYGPGGEGPGGAGGPYGPGGVG
PGGSGPGGYGPGGSGPGGYGPGGAGPGGYGPGGSGPGGYGPGGSGPGGYGPGGSGPGGY
GPGGSGPGGYGSGGAGPGGYGPGGSGPGGYGPGGSGPGGYGPGGTGPGGTGPGGSGPGG
YGPGGSGPGGSGPGGSGPGGYGPSGSGPGGYGPSGSGPGGYGPGGSGPGGYGPGGSGAG
GTGPGGAGGAGGAGGSGGAGGSGGAGGSGGAGGSGGVGGSGGTTITEDLDITIDGADGP
ITISEELTIS

26) *N.i.* Flag
GGAGAGGVGPGGSGPGGVGPGGSGPGGVGPGGSGPGGVGSGGSGPGGVGPGGSGPGGVG
SGGFGPGGIGPGGSGPGGVGPGGVGGPYGPGGSGPGGAGGAGGSYGPGGPYGPGGSGGP
GGAGGPYGPGGAGGPYGPGGPYGPGGAGGPGGEGPGGAGGPYGPGGPGGAGPGGYGPGG
AGPGGYGPGGAGPGGYGPGGAGSGGYGPGGAGPGGYGPGGPGPGGYGPGGAGPGGYGPG
GTGPGGSAPGGAGPGGAGPGGYGPGGSGPGGYGPGGGPGGAGPGGAGPGGAGPGGAGPG
GAGPGGAGPGGAGPGGAGPGGAGPGGAGPGGVGTGGLGRGGAGRGGAGRGGAGRGGAGR
GGAGRGGTGGVGGAGGAGGAGGVGGAGGSGGTTVIEDLDITIDGADGPITISEELTI
