# Supplementary material for: Untangling spider silk evolution with spidroin terminal domains
Source: BMC Evol Biol. 2010 Aug 9;10:243. doi: 10.1186/1471-2148-10-243 (PMC2928236; doi:10.1186/1471-2148-10-243)
Supplement: Additional file 7 — Presence or absence of structural motifs in spidroin exemplar repeats. 0 = absent, 1 = present. [file 1471-2148-10-243-S7.PDF]

Additional file 7. Presence or absence of structural motifs in spidroin exemplar repeats. 0=absent, 1=present.

	A_n	$GPG(X)_n$	$(GGX)_n$	$(GA)_n$	$(GS)_n$
N.c. MaSp1a	1	0	1	1	0
N.c. MaSp1b	1	0	1	1	0
N.c. MaSp2	1	1	0	0	0
L.h. MaSp1	1	0	1	1	0
L.h. MaSp2	1	1	0	0	0
L.g. MaSp1	1	0	1	0	0
N.i. MaSp2	1	1	0	0	0
A.t. MaSp2	1	1	0	0	0
E.a. MaSp	1	0	1	0	0
*L.h.*TuSp1	0	0	0	0	0
A.a TuSp1	0	0	0	0	0
N.i. Flag	0	1	1	1	0
*B.c.*fibroin1	1	0	0	0	0
*D.c.*MaSp	1	0	0	1	1
*D.c.*MaSp-like	1	0	1	1	0
*D.s.*MaSp2	1	1	1	0	0
K.h. MaSp1	1	0	1	1	1
L.h. MiSp	0	0	1	1	1
A.ap. TuSp1	0	0	0	0	0
A.ap. MaSp	0	0	0	1	0
M.g. MiSp	1	0	1	1	1
U.d. MiSp	1	0	0	1	1
A.v. Flag	0	1	1	1	0
A.b. TuSp1	0	0	0	0	0
N.c. Flag	0	1	1	0	0
N.ct. TuSp1	0	0	0	1	0
